# Autoptic Findings in Patients Treated with (VA-ECMO) after Cardiac Arrest

**DOI:** 10.3390/diseases12100245

**Published:** 2024-10-07

**Authors:** Martina Focardi, Francesco Santori, Beatrice Defraia, Rossella Grifoni, Valentina Gori, Ilenia Bianchi, Manuela Bonizzoli, Chiara Lazzeri, Adriano Peris

**Affiliations:** 1 Forensic Pathology Unit, AOU Careggi, Largo Brambilla 3, 50134 Florence, Italy; martina.focardi@unifi.it (M.F.); beatrice.defraia@unifi.it (B.D.); rossella.grifoni@unifi.it (R.G.); 2 Forensic Medical Sciences, Department of Health Science, University of Florence, Largo Brambilla 3, 50134 Florence, Italy; francesco.santori@unifi.it (F.S.); valentina.gori@unifi.it (V.G.); 3 Laboratory of Personal Identification and Forensic Morphology, Department of Health Sciences, University of Florence, Largo Brambilla 3, 50134 Florence, Italy; 4 Intensive Care Unit and Regional ECMO Referral Centre, AOU Careggi, Largo Brambilla 3, 50134 Florence, Italy; manuelabonizzoli@gmail.com (M.B.); lazzeric@aou-careggi.toscana.it (C.L.); adriano.peris@gmail.com (A.P.)

**Keywords:** ECMO, ischemic complications, autopsy findings

## Abstract

**Background:** This study examines the results of autopsy examinations specifically aimed at documenting complications arising from the implantation phase and treatment with veno–arterial extracorporeal membrane oxygenation (VA-ECMO) in patients with refractory cardiac arrest. ECMO and VA-ECMO in particular are life-saving interventions that, in the case of cardiac arrest, can temporarily replace cardiac pump function. VA-ECMO is, however, a very invasive procedure and is associated with early mechanical, haemorrhagic, and thrombotic events, infections, and late multi-organ dysfunction. **Aim:** This research aims to evaluate autoptic and histologic findings in patients on VA-ECMO support, providing clinical and forensic evaluation elements with respect to the procedure and clinical settings. **Materials and Methods:** The study analysed 10 cases, considering variables such as the duration of cardiac arrest, understood as the time between the cardiac arrest event and reperfusion with VA-ECMO, the duration of VA-ECMO support, and any complications detected by clinicians during treatment. **Results:** The results highlighted the presence of numerous ischemic and haemorrhagic events affecting various organs. Among them, the intestines were particularly vulnerable, even after a short ECMO duration. **Conclusions**: ECMO was found to accelerate post-mortem decomposition, affecting post-mortem interval estimations, and cardiac damage from reperfusion, underlining the need to meticulously select indications for treatment with VA-ECMO and perform constant clinical evaluations during the treatment itself.

## 1. Introduction

ECMO is a critical intervention in the management of patients with severe cardiopulmonary dysfunction [1]. This extracorporeal technique of supporting organ perfusion facilitates adequate gas exchange and circulatory support in critical situations, allowing for the potential recovery of otherwise compromised patients. However, despite its significant benefits, veno–arterial ECMO is not devoid of potential complications, which are often underreported [2]. These complications, ranging from mechanical, haemorrhagic, and thrombotic events to increased incidence of infection, can have substantial consequences for patient health, necessitating accurate clinical evaluation and management [3]. Prolonged ECMO therapy is often associated with a systemic inflammatory reaction, persistent vasoplegia, and coagulopathy, which can contribute to multiple organ failure.

This article aims to offer a dual perspective for both forensic and clinical practitioners to assess, by means of an autopsy, ischemic complications in patients with refractory cardiac arrest treated with veno–arterial ECMO. In the recent scientific literature, there has been an impressive diffusion of the ECMO technique in tertiary centres; however, reports of autopsy studies are practically missing, even in systematic meta-analyses [4]. Available evidence on histopathologic findings in VA-ECMO is scarce and heterogeneous due to different inclusion criteria for the study population and inconsistent numbers [5]. Most investigations have focused on histological changes due to ECMO treatment. Histopathologic findings across various organs in patients treated with ECMO are poorly understood. Therefore, our goal was to investigate histopathological findings in multiple organs of patients treated with VA-ECMO and describe our experience of autoptic findings in patients managed with ECMO.

Bedside extracorporeal circulation techniques currently have three main indicators:

- VV-ECMO is primarily indicated for patients with severe respiratory failure but relatively preserved cardiac function. This modality is used to ensure adequate oxygenation and carbon dioxide removal from the blood in cases of acute respiratory distress syndrome (ARDS), severe pneumonia, or other critical pulmonary conditions. Patients requiring VV ECMO are typically those who do not respond to conventional therapies, such as advanced mechanical ventilation, and need prolonged support to allow their lungs to recover [6];
- VA-ECMO is reserved for patients with acute cardiac failure, which may or may not be associated with respiratory failure [7]. It is often used in situations of refractory cardiac arrest, cardiogenic shock, or other critical conditions where both circulatory and respiratory support are necessary. VA-ECMO is used for patients with severely compromised cardiac function, who require immediate and temporary support for systemic circulation and oxygenation [8];
- Extracorporeal Interval Support for Organ Retrieval (EISOR) is a modality of extracorporeal circulation that selectively targets splanchnic organs for organ transplantation using the same vascular access as VA-ECMO [9];
- Donative ECMO: In cases where a patient on VA-ECMO does not respond to therapies and is declared brain dead, organ donation via ECMO can be considered. Additionally, for patients with non-treatable cardiac arrest, ECMO can be used as temporary support in a controlled non-heart-beating donation (uDCD) protocol [10]. This approach maintains organ perfusion until transplant evaluation. Selection for donative ECMO requires rigorous clinical and ethical assessment, with families actively involved in the decision-making process [11]. The clinical significance of autopsy findings in these patients may be twofold. Firstly, the identification of mechanical and ischemic complications related to ECMO support may help clinicians tailor and modify treatment. Secondly, among patients on ECMO support, some may develop brain death and become donors after brain death, while others without therapeutic options and with consent to donation may become uncontrolled donors after circulatory death. In this context, autopsy findings can help assess organ viability and determine their suitability for transplantation.

We present a case series including a comprehensive review of the literature to identify and categorise complications associated with ECMO support.

## 2. Materials and Methods

To investigate the complications associated with VA-ECMO procedures in patients affected by refractory cardiac arrest at the ECMO Center of Careggi Hospital (Florence, Italy), this study examined 10 cases of complete autopsies and histological examinations in the Section of Legal Medicine at Careggi Hospital (Florence, Italy).

From 2019 to 2023, 154 patients with out-hospital cardiac arrest (OHCA) were admitted to the Regional ECMO Center of Careggi Teaching Hospital. In 61 out of 154 patients (40%), restoration of spontaneous circulation was achieved (ROSC). Among cardiac arrest patients without ROSC (N.93), ECPR was implanted in 44 (47%) patients considered to be in refractory cardiac arrest and, therefore, potential responders to ECPR.

Of the 34 patients treated with ECPR, 6 patients survived (17%, 6/34) with good neurological outcomes, whereas 28 patients died (28/34, 82%). Among those who died, 12 were potential donors (42%, 12/28). Of the 28 patients affected by sudden cardiac death who died after VA-ECMO implantation, 10 patients underwent autopsy studies.

Careggi Teaching Hospital, a tertiary-level university hospital, applies a protocol to manage patients requiring ECMO. The hospital follows stringent clinical guidelines [12] and ethical considerations to ensure the best outcomes for patients and facilitate organ donation when possible.

The emergency medical system alerts the physician in charge of the emergency department in all cases of witnessed cardiac arrest from the perspective of a therapeutic fast track or donation path. A certified ECMO team (including an intensivist, a cardiac surgeon, a cardiologist, and a perfusionist) is immediately activated. In the case that all criteria (Table 1) are met, therapeutic ECMO may be attempted to sustain the subject’s life through proper reperfusion. To perform ECMO, correct anticoagulation must be achieved through the administration of unfractionated heparin, targeting an activated partial thromboplastin time (aPTT) of 50–70 s. Furthermore, vasoactive drugs, such as norepinephrine and vasopressin, were administered as needed to maintain a mean arterial pressure (MAP) above 60 mmHg, ensuring adequate circulatory support throughout the ECMO procedure.

The study, approved by the Internal Board, involved the autopsy documentation of mechanical, haemorrhagic, and ischemic complications resulting from the cannulation phase for starting VA-ECMO and those relating to extracorporeal oxygenation treatment. In particular, the vascular structures affected by cannulation and the macro and microscopic alterations at the cerebral, pulmonary, renal, and intestinal levels were considered.

This study also considered, as critical elements of damage assessment, the duration of cardiac arrest before the start of perfusion with VA-ECMO and the time of treatment with VA-ECMO before death. We conducted descriptive statistical analyses to summarise and interpret the data collected.

A VA-ECMO protocol operating in our hospital includes a therapeutic arm for treating refractory cardiac arrest and a donor arm (uncontrolled DCD) for the transplantation of splanchnic organs. In a uDCD protocol, if irreversible circulatory death is certified, local procurement coordination is involved. If all inclusion criteria are fulfilled, the patient is recognised as a potential donor.

The enrolment protocol included the VA-ECMO procedure and the uDCD protocol (Table 1). In all cases cardiac arrest was witnessed, patients were identified and ECMO started (from cardiac arrest) <90 min. To declare death, a 20-min no-touch period is required by Italian legislation with continuous electrocardiographic recording of the absence of any cardiac electrical activity. During this period, the family is informed of the death and organ donation possibility. If the family consents, normothermic regional perfusion (NRP) begins with femoral artery cannulation and donative ECMO support. 

## 3. Results

Based on the inclusion criteria, we identified 10 cases (Table 2). The age range was 18 to 65 years with a mean age of 44. Among the patients, there were 7 males and 3 females. The average duration of cardiac arrest among our cases was 58 min, and the average duration of ECMO support was 257.4 min. The duration of cardiac arrest ranged from 45 to 122 min. Despite differences in cardiac arrest and ECMO duration, intestinal ischemic lesions were present in all patients except two (8/10, 80%).

Encephalic parenchymal congestions were observed only in two patients, not related to cardiac arrest and/or ECMO duration. Microscopic exam documented haemorrhagic lesions in patients with no macroscopic alterations.

In half cases haemorrhagic complications were detected in lungs, confirmed at microscopic examination.

Haemorrhagic lesions were detected in kidneys at macroscopic examination in half cases, while congestions and haemorrhagic spots were macroscopically detected in two cases, respectively.

Cadaveric transformative phenomena appeared accelerated compared to expected postmortem timelines, particularly regarding the progression of putrefactive changes such as the development of green putrefactive staining, notably in one case involving a young athlete.

## 4. Discussion

The utilization of ExtraCorporeal Membrane Oxygenation (ECMO) is a critical intervention in the management of patients with severe cardiopulmonary dysfunction [1]. This extracorporeal technique facilitates adequate gas exchange and circulatory support in critical situations, allowing for the potential recovery of otherwise compromised patients. However, despite its significant benefits, ECMO is not devoid of potential complications, which are often underreported [2,3,13]. These complications, albeit rare, can have substantial repercussions on patient health, necessitating meticulous clinical evaluation and management. In refractory cardiac arrest cases that are not suitable for therapeutic ECMO, uncontrolled DCD may be considered to allow these patients (and their families) to donate [14].

The novelty of the present case series is represented by the study population, including patients treated with VA-ECMO for refractory cardiac arrest and donors after uncontrolled DCD. Evidence on this topic is scarce, especially in cases involving complete autopsies and histological exams, with heterogeneous findings due to discrepancies in study populations. This is a rare investigation including uDCD, which is characterised by a shorter duration of ECMO supported by a longer duration of cardiac arrest. Findings in these patients have clinical relevance in transplantation medicine.

Previous studies, all performed on ECMO patients, have identified discrepancies between clinical and postmortem examinations and primarily focused on assessing ECMO complications. Rastan et al. reported clinical and postmortem data in 154 patients who died after postcardiotomy, documenting an underestimation of thromboembolic events [15]. Cho et al. presented a retrospective cohort study of adult ECMO patients with brain autopsies, finding acute brain injuries not clearly attributable to ECMO support but possibly related to the primary pathology (e.g., cardiac arrest, shock states) [16]. Trejnowska et al. conducted a retrospective investigation of lung autopsies from VV and VA-ECMO recipients, concluding that histopathologic findings were heterogeneous due to different inclusion criteria [17].

Lee et al. studied the autopsied lungs of ECMO patients and found that pulmonary haemorrhage and acute lung injury, common histomorphologic findings, were more prevalent in ECMO-treated patients than non-ECMO patients [18].

In Table 3, we reported a literature review [6,19,20,21,22,23,24,25,26] about the major complications.

Our case series differs from previous investigations [26] for two reasons: the study population includes uDCD and the short duration of ECMO support. The latter may account for the fact that we did not observe macroscopic intracranial haemorrhages, frequently encountered in previous investigations. By contrast, histological examination revealed haemorrhages in one out of ten cases and oedema in five out of ten cases.

In the literature, neurological complications associated with ECMO range from 8% to 50%. Specifically, haemorrhagic complications are reported in 2.8–21% of cases and are associated with a worsened prognosis. Intracranial haemorrhages are the most common, followed by subarachnoid haemorrhages [27]. These complications may be related to heparin treatment, beyond the underlying clinical conditions.

Regarding potential myocardial ischemia, macroscopic examination identified three cases with early infarction signs such as pallor. Histological analysis identified reperfusion injury in two cases, distinguishable from primary myocardial damage. Additionally, myocardial ischemia was evident in three cases, with small hemorrhages identified in three out of ten cases. These data strongly suggest that cardiac causes of death may be detectable even under ECMO treatment since it is a supportive therapy. In Case 1 and Case 10, histological analysis revealed significant myocardial damage consistent with reperfusion injury. This case presented with myocardial pallor, scarring, and fat infiltration, indicating both recent and old myocardial infarction. The histological findings included scattered myocardial necrosis and preserved architecture with signs of reperfusion injury. These findings underscore the necessity of careful histological evaluation in autopsies of ECMO patients to differentiate between reperfusion injury and primary cardiac pathology. Such differentiation is crucial for accurately determining the cause of death and understanding the extent of myocardial damage due to ECMO-related interventions. We also observed that cases with the longest duration of ECMO (Cases 1, 9, and 10) were those with the most implications and where cardiac reperfusion injury occurred in two out of three cases.

Pulmonary haemorrhages were observed in seven out of ten cases. Moreover, in the lungs, four out of ten cases showed pulmonary inflammation, which, according to the literature, predisposes to Diffuse Alveolar Damage (DAD). The study identified prevalent ischemic damage in the lungs. Pulmonary ischemia was characterised by significant alveolar damage and haemorrhagic infarctions. Our findings may be of interest to transplantation medicine since uDCD-only lung programs are being implemented in many countries. Due to peculiar lung physiology, lung transplantation from uDCD shows promising results. The detection of haemorrhagic lesions can be related both to heparin treatment and mechanical chest devices used for chest compression.

Organs from uDCD undergo further assessment before transplantation by ex vivo machine perfusion to assess organ viability and repair the ischemic perfusion injury.

Intestinal ischemia presented with extensive mucosal necrosis and haemorrhage in seven out of ten cases, corroborating the data presented in the literature [28]. These findings underscore these organs’ vulnerability to ischemic injury during ECMO support. Intestinal ischemia also presented with extensive mucosal necrosis and haemorrhage, which was particularly evident in Case 3, where the intestines were blackened from the duodenum to the colon, indicating widespread necrosis. Similarly, Case 9 showed congested and oedematous mucosa with focal areas of necrosis, reinforcing the intestines’ vulnerability. The microscopic analysis of these cases often revealed extensive mucosal and submucosal necrosis with haemorrhagic infiltration, underscoring the severe ischemic impact on the gastrointestinal tract during ECMO support. Interestingly, intestinal ischemia, detected in the majority of patients, can be considered quite an early complication considering the ECMO duration in our series.

In our series, it was not possible to detect signs of infection as the subjects died shortly after the procedure, and did not have enough time to manifest this complication. Similarly, the rapid progression of their condition precluded the observation of other potential inflammatory responses typically associated with prolonged ECMO therapy.

These findings highlight the critical need for early and aggressive management strategies to mitigate these severe complications and improve patient outcomes.

Summary of Key Findings

- Ischemic Complications: Intestines are particularly vulnerable, even after a short ECMO duration;
- Postmortem Changes: ECMO appears to accelerate postmortem decomposition, complicating traditional post-mortem interval estimations;
- Myocardial Reperfusion Injury: Histological analysis is essential to distinguish between reperfusion injury and primary cardiac pathology in ECMO patients.

These findings collectively emphasise the complex pathological landscape associated with ECMO support and highlight the need for specialised autopsy protocols and histological evaluations in these patients.

The analysis conducted provides valuable insights into the nature and complications associated with ECMO use in CPA patients. The heterogeneity of the causes of death reflects the complexity of the cases studied; nonetheless, a recurring pattern of intestinal ischemia was identified. The study also highlights the pulmonary component, which is underrepresented in the literature. The observed accelerated postmortem decomposition suggests the ECMO procedure’s potential impact on the progression of transformative cadaveric phenomena.

This study has several limitations that must be acknowledged. Firstly, the sample size was limited to only 10 cases, which restricted the generalizability of the findings. Additionally, the retrospective nature of the study allowed for the analysis of a limited number of variables, constraining the depth and breadth of the findings.

The results of our study have significant implications for both forensic and clinical practice. For forensic practitioners, the identification of reperfusion injury and accelerated decomposition necessitates a thorough and nuanced approach to autopsy examinations in ECMO patients, considering the duration of cardiac arrest and ECMO support as important factors. For clinicians, the direct correlation between ischemic damage, the duration of cardiac arrest, and the timing of implantation is pivotal.

Further research is required to deepen our understanding of ECMO-related complications, particularly ischemic damage, and refine autopsy protocols to better identify and interpret these findings. Additionally, prospective studies investigating the impact of ECMO on postmortem changes would be beneficial. Enhanced interdisciplinary collaboration between forensic pathologists and clinicians will be crucial to advancing the field and improving patient care.

## 5. Conclusions

This research focuses on the complexities and challenges associated with ECMO use in CPA patients. It highlights the need for meticulous risk–benefit assessments and targeted clinical management to optimise outcomes. The identification of ischemic and reperfusion complications provides a foundation for future studies aimed at improving our understanding of ECMO-related complications and developing more effective preventive and therapeutic strategies. The duration of cardiac arrest and ECMO support should be considered for forensic purposes.

## Figures and Tables

**Table 1:** Sample inclusion criteria for the VA-ECMO procedure and uDCD protocol.

Inclusion Criteria for Therapeutic VA-ECMO	Inclusion Criteria for uDCD Protocol
age 15–65 years	age 18–65 years
witnessed cardiac arrest	witnessed cardiac arrest and clear patient identification
no flow time < 5 min	no flow time < 5 min
cardiac arrest hospital time < 90 min	cardiac arrest hospital time < 90 min
shockable rhythm	cardiac arrest—the 20-min no touch time < 150 min

**Table 2:** Case series dataset.

	Case 1	Case 2	Case 3	Case 4	Case 5	Case 6	Case 7	Case 8	Case 9	Case 10
**Intestinal Findings:**	Mucosa with reddish spots	Intestinal blackish, (sign of necrosis)	Blackish intestines (duodenum to colon)	No evidence	Haemorrhagic mucosa, ischemic suffering	Necrosis, haemorrhages	No evidence	Blackish mucosa, inflammation	Focal Necrosis	Haemorrhages, focal necrosis
**Encephalic Findings:**	No evidence	No evidence	No evidence	No evidence	No evidence	No evidence	Parenchymal congestion	Parenchymal congestion	Parenchymal congestion	No evidence
**Cardiac Findings:**	Myocardial pallor	Scarring, pale myocardium.	No evidence	No evidence	No evidence	No evidence	Multiple embolic formations	Haemorrhagic spots	Myocardial pallor, and fibrosis	LV Hypertrophy Focal areas of pallor
**Pulmonary Findings:**	Anthracotic pattern, enlarged lymph nodes	No evidence.	Collapsed lungs, subpleural petechiae	Petechiae	Black speckled pattern, petechiae	No evidence	Numerous petechiae	No evidence.	Petechial haemorrhages	Petechiae
**Kidney Findings:**	Urinous cyst	No evidence.	No evidence	Parenchymal congestion	No evidence	No evidence	No evidence	No evidence.	No evidence	Hemorrhagic spots
**Micro Intestinal:**	Ischemic necrosis, inflammatory. infiltrates, hemorrhages	Necrosis spots	Extensive necrosis and inflammation	No evidence	Haemorrhagic infiltration	Haemorrhagic infiltration, mucosal erosion	No evidence	Haemorrhagic infiltration.	Extensive mucosal necrosis, haemorrhagic infiltration.	Haemorrhages
**Micro Cardiac:**	Myocardial necrosis with signs of reperfusion injury	No evidence	No evidence	Minimal haemorrhages	Dense inflammatory infiltration, necrosis	No evidence	No evidence	Inflammation, myocardial haemorrhage	Myocardial fibrosis, inflammatory cells	Myocardial necrosis, reperfusion injury
**Micro Pulmonary**	Haemorrhagic infarction, chronic inflammation	No evidenc.	Inflammation, haemorrhagic areas	Emphysema, alveolar haemorrhage	Alveolar haemorrhage	No evidence	Inflammation, hemorrhagic infarction	No evidence	Alveolar haemorrhage, interstitial fibrosis, emphysema	Haemorrhage interstitial, inflammatory cells
**Micro Encephalic**	Minimal vasogenic oedema	No evidence	No evidence	No evidence	Oedema, spot of haemorrhagic lesions	No evidence	No evidence	Congestion, no ischemic lesions	No evidence	Not evidences. perivascular and interstitial oedema
**Micro Kidney:**	Multiple and focal hemorrhagic areas	No evidence	No evidence	No evidences	No evidence	No evidence	Haemorrhagic areas	Inflammatory infiltrate	Haemorrhagic areas	Haemorrhagic spots
**ECMO Duration:**	24 h	1 h 57 min	3 h 1 min	2 h 7 min	1 h 7 min	2 h 1 min	1 h 36 min	35 min	4 h	2 h 30 min
ACR Duration *	45 min	122 min	66 min	50 min	27 min	51 min	41 min	32 min	71 min	49 min

* (time between the onset of cardiac arrest and reperfusion with VA-ECMO).

**Table 3:** ECMO-associated complications.

Type of Complication	Frequency	Causes	Reference
**Intracranial Haemorrhage**	Significant percentage of ECMO patients	Use of anticoagulants to prevent coagulation in the ECMO circuit	Lüsebrink E et al., 2022 [19]
**Pulmonary Haemorrhage**	Common in ECMO patients	Systemic anticoagulation and vascular damage	Turner DA et al., 2013 [20]
**Nosocomial Infections**	High incidence of ventilator-associated pneumonia	Prolonged use of catheters and medical devices	Mornese Pinna S et al., 2023 [21]
**Myocardial Damage**	Can occur despite ECMO support	Primary disease or ECMO-related complications	Paddock S et al., 2024 [22]
**Diffuse Alveolar Damage (DAD)**	Common in ECMO patients for ARDS	Oedema, inflammatory infiltrate, hyaline membrane	Alessandri F et al., 2023 [23]
**Ventilation-related Complications**	Common due to mechanical ventilation	High lung pressures	Brodie D et al., 2011 [6]
**Acute Kidney Injury**	Frequently observed in ECMO patients	Hypoperfusion, sepsis, use of contrast agents	Zwiers AJ et al., 2013 [24]
**Subarachnoid Haemorrhage**	Rare	Anticoagulation and vascular fragility	Fletcher-Sandersjöö A et al., 2017 [25]
**Intestinal Ischemia and Necrosis**	Common in ECMO patients	Hypoperfusion, microthrombosis	Cavayas YA et al., 2018 [26]

## Data Availability

The original contributions presented in the study are included in the article, further inquiries can be directed to the corresponding author.
